# Risk Factors for PVC Induced Cardiomyopathy and Post-Ablation Left Ventricular Systolic Dysfunction Reversibility: A Systematic Review and Meta-Analysis of Observational Studies

**DOI:** 10.31083/j.rcm2509327

**Published:** 2024-09-11

**Authors:** Dongsheng Zhao, Qiushi Chen, Zhongyin Zhou, Pengcheng Zhao, Jianzhou Shi, Jun Yin, Qing Zhang, Fengxiang Zhang

**Affiliations:** ^1^Department of Cardiology, The Second Affiliated Hospital of Nantong University, 226001 Nantong, Jiangsu, China; ^2^Department of Cardiology, The First Affiliated Hospital of Nanjing Medical University, 210029 Nanjing, Jiangsu, China; ^3^Department of Cardiology, Children's Hospital of Nanjing Medical University, 210093 Nanjing, Jiangsu, China

**Keywords:** premature ventricular complexes, left ventricular systolic dysfunction, risk factors, meta-analysis

## Abstract

**Background:**

Premature ventricular complex (PVC) induced cardiomyopathy (PVC-CMP) and exacerbated left ventricular systolic dysfunction (LVSD) are common in clinical scenarios. However, their precise risk factors are currently unclear.

**Methods:**

We performed a systematic review of PubMed, EMBASE, Web of Science, and Chinese-based literature database (CBM) to identify observational studies describing the factors associated with PVC-CMP and post-ablation LVSD reversibility. A total of 25 and 12 studies, involving 4863 and 884 subjects, respectively, were eligible. We calculated pooled multifactorial odds ratios (OR) and 95% confidence intervals (CI) for each parameter using random-effects and fixed-effects models.

**Results:**

The results showed that 3 independent risk factors were associated with PVC-CMP: being asymptomatic (OR and 95% CI: 3.04 [2.13, 4.34]), interpolation (OR and 95% CI: 2.47 [1.25, 4.92]), and epicardial origin (epi-origin) (OR and 95% CI: 3.04 [2.13, 4.34]). Additionally, 2 factors were significantly correlated with post-ablation LVSD reversibility: sinus QRS wave duration (QRSd) (OR and 95% CI: 0.95 [0.93, 0.97]) and PVC burden (OR and 95% CI: 1.09 [0.97, 1.23]).

**Conclusions:**

the relatively consistent independent risk factors for PVC-CMP and post-ablation LVSD reversibility are asymptomatic status, interpolation, epicardial origin, PVC burden, and sinus QRS duration, respectively.

## 1. Introduction

Premature ventricular complexes (PVCs) and left ventricular systolic dysfunction (LVSD) often coexist in clinical practice. Until 2016, PVC was recognized as a reversible cause of dilated cardiomyopathy, known as PVC-induced cardiomyopathy (PVC-CMP) [1]. Despite numerous efforts to investigate predictors of PVC-CMP [2, 3, 4, 5], it remains challenging to differentiate PVC-CMP from other types of dilated cardiomyopathy complicated by frequent PVC. Furthermore, it is unclear why some patients with frequent PVC develop cardiomyopathy while others do not [6, 7, 8]. Identifying which patients and when PVC elimination is needed to promote cardiac reverse remodeling, which remains an unresolved issue. Therefore, we conducted a systematic review and meta-analysis to evaluate the risk factors for PVC-CMP and the reversibility of LVSD following ablation, with or without a definitive etiology.

## 2. Methods

We conducted a systematic review and meta-analysis following the Meta-analysis of Observational Studies in Epidemiology (MOOSE) protocol [9] throughout the study’s design, implementation, analysis, and reporting. We did not find related randomized controlled trials (RCTs) available at the time, so we did not register the study.

### 2.1 Search Strategy and Study Selection 

We systematically searched 3 English-based literature databases (PubMed, Embase, and Web of Science) and 1 Chinese-based literature database (CBM) for cross-sectional studies, case-control studies, and cohort studies from January 1990 to July 2021. We used subject headings (MeSH and Emtree) and text words related to PVC and LVSD. The full literature search strategy is presented in **Supplementary Material 1**.

Studies meeting the following criteria were eligible: (1) published in a peer-reviewed journal in English or Chinese; (2) reported correlative factors of PVC-CMP (compared with PVC patients without LVSD) or post-ablation LVSD reversibility; (3) reported odds ratios (OR) and 95% confidence intervals (CI) (or provided sufficient data for calculation). We excluded studies involving cardiac resynchronization therapy (CRT) and acute myocardial injuries, such as acute myocardial infarction, myocarditis, and Takotsubo cardiomyopathy. 2 investigators independently assessed the eligibility of each study, resolving disagreements through consensus.

### 2.2 Data Extraction and Quality Assessment

Data extraction was completed by 2 investigators and checked by 2 others using pre-designed forms (**Supplementary Material 2**). Extracted data included the first author, year of publication, journal, study design, study location, inclusion and exclusion criteria, cohort size, multivariate analysis model, variable inclusion criteria, and OR and 95% CI for each variable and covariate included in the statistical models.

Quality assessment was carried out using the Newcastle-Ottawa Scale (NOS) for cohort studies [10]. 3 items about the selection, comparability, and outcome were checked. A score of ≥7 was considered good quality [11]. The Cross-Sectional/Prevalence Study Quality tool was used for cross-sectional study quality assessment. 11 items were used; an item received a score of “1” if the answer was “yes” and a score of “0” if the answer was “no” or “unclear”. Scores of 0–3, 4–7, and 8–11 points were classified as “low quality”, “moderate quality”, and “high quality” respectively [12] (**Supplementary Material 2**).

### 2.3 Data Synthesis and Analysis

We combined the OR and corresponding 95% CI for each variable obtained through multivariate analysis. To minimize reporting bias, we conducted meta-analyses only for factors with multifactorial risk estimates reported in more than half of the studies.

We used StataSE (version 16.0, StataCorp, College Station, TX, USA) to conduct the meta-analyses (Meta forestplot) and assess potential publication bias (Meta funnelplot). Risk estimates were calculated using the generalized least squares method by assuming linearity of the natural log-scale ORs. Cochran’s Q statistic and I^2^ statistic were used to quantify heterogeneity among included studies. For outcomes with high heterogeneity (I^2^ > 50%), we used the random-effects model, while outcomes with low and moderate heterogeneity (I^2^ ≤ 50%) employed the fixed-effects model. We evaluated publication bias using Begg’s test.

## 3. Results

### 3.1 Characteristics of Eligible Studies

**Fig. 1.**
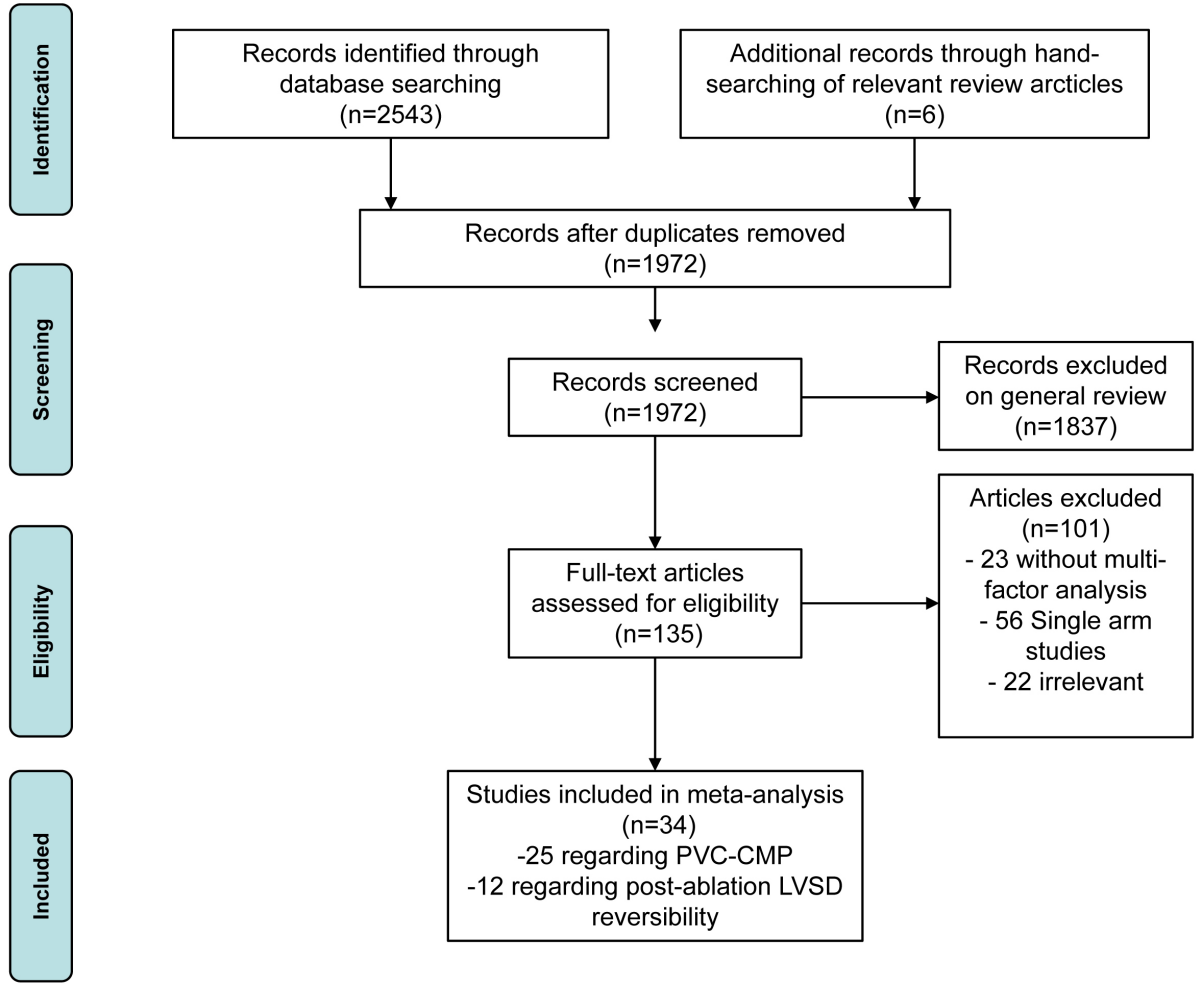
**Preferred Reporting Items for Systematic Reviews and Meta-Analyses (PRISMA) flow diagram**. PVC-CMP, premature ventricular complexes induced cardiomyopathy; LVSD, left ventricular systolic dysfunction.

We screened 2553 articles, of which 587 duplicates and 1835 articles with titles or abstracts were excluded. After a thorough review of the remaining 97 papers, 34 articles were included for further analysis (Fig. 1). The characteristics of eligible studies are summarized in Table 1 (Ref. [2, 3, 4, 5, 6, 13, 14, 15, 16, 17, 18, 19, 20, 21, 22, 23, 24, 25, 26, 27, 28, 29, 30, 31, 32, 33, 34, 35, 36, 37, 38, 39, 40]). Among them, 25 studies, involving 4863 subjects, focused on PVC-CMP [2, 3, 4, 5, 13, 14, 15, 16, 17, 18, 19, 20, 21, 22, 23, 24, 25, 26, 27, 28, 29, 30, 31, 32, 33], while 12 studies, involving 884 subjects, addressed factors related to post-ablation LVSD reversibility [6, 17, 26, 31, 33, 34, 35, 36, 37, 38, 39, 40].

**Table 1.** **Summary of cohorts contributing data to systematic review**.

First author, year	Journal	Study type	Country/region	Inclusion criteria	Exclusion criteria	Cohort size	Observe events
Koca H, 2020 [31]	Pace-Pacing and Clinical Electrophysiology	cross-sectional	Turkey	symptomatic PVCs scheduled for RFCA	SHD/PVC recurrence	150	LVEF <50%
Krishnan B, 2017 [26]	JACC Clin Electrophysiol	cohort	America	PVC >10%/24 h underwent successful ablation	known cause of LVD	61	LVEF <50%
Latchamsetty R, 2015 [3]	JACC Clin Electrophysiol	retrospective cohort	America; Germany	RFCA for frequent idiopathic PVCs	prior infarcts or delayed enhancement identified by CMRI	1185	LVEF <50%
Lee A, 2019 [29]	Heart Lung Circ	retrospective cohort	Australia	patients for ablation of PVCs	with pre-existing scar substrate	152	LVEF <50%
Mao J, 2021 [33]	Sci Rep	retrospective cohort	America	undergone PVCs ablation	CAD/VHD/ARVC and cardiac sarcoidosis	51	LVEF <45%
Niwano S, 2009 [14]	Heart	prospective cohort	Japan	frequent OT PVCs (>1000/day)	any detectable heart disease	239	∆LVEF >–6%
Olgun H, 2011 [16]	Heart Rhythm	cross section	America	frequent PVCs referred for catheter ablation	/	51	LVEF <50%
Park KM, 2017 [4]	Int J Cardiol	Bidirectional cohort	Korea	symptomatic frequent PVCs (>10% PVCs per 24 h)	SHD	144	LVEF <50%
Parreira L, 2019 [30]	Cardiology Research	retrospective cohort	Portugal	frequent PVCs (>1% PVCs )	history of documented AF or AFL	285	HF end point
Sadron Blaye-Felice M, 2016 [5]	Heart Rhythm	retrospective cohort	France; Switzerland	patients referred for PVC ablation	frequent nonsustained VT	168	LVEF <50%
Voskoboinik A, 2020 [2]	Heart Rhythm	cross section	America	PVCs (average daily burden >5%)	SHD	206	LVEF <45%
Yamada S, 2018 [27]	J Interv Card Electrophysiol	cross section	Taiwan	RVOT PVCs undergoing ablation	SHD/DCM	130	LVEF <50%
Yokokawa M, 2012 [18]	Heart Rhythm	retrospective cohort	America	PVCs undergoing ablation	SHD	294	LVEF <50% or ∆LVEF >10%
Ban JE, 2013 [19]	Europace	cross section	Korea	frequent PVCs (>10% per 24 h)	SHD	127	LVEF <50%
Kanei Y, 2008 [13]	Ann Noninvasive Electrocardiol	cross section	Japan	frequent (≥10 PVCs per hour) RVOT PVCs	IHD, SHD, or other cause of LVSD	108	LVEF <55%/45% (SPECT)
Billet S, 2019 [28]	Heart Rhythm	retrospective cohort	France	frequent PVCs referred for catheter ablation	frequent non-sustained VT (>1% QRS complexes)	33	LVEF <50%
Kawamura M, 2014 [21]	J Cardiovasc Electrophysiol	prospective cohort	America	undergoing successful ablation of PVCs	other causes of cardiomyopathy	214	LVEF <50%
Hamon D, 2016 [24]	J Cardiovasc Electrophysiol	prospective cohort	America; France	frequent PVCs (≥5%) referred for ablation	/	102	LVEF <50%
Ghannam M, 2021 [32]	Heart Rhythm	retrospective cohort	America; France	frequent PVCs referred for ablation	/	351	LVEF <50%
Carballeira Pol L, 2014 [20]	Heart Rhythm	prospective cohort	America; France	>10% PVCs with normal LVEF	structural or genetic heart disease	45	LVEF <50%/∆LVEF >–10%
Bas HD, 2016 [23]	Heart Rhythm	retrospective cohort	America	frequent PVCs referred for catheter ablation	SHD	107	LVEF <50%
Deyell MW, 2012 [17]	Heart Rhythm	retrospective cohort	America	patients with successful ablation of PVCs	/	103	LVEF <50%
Yifan Mu, 2015 [22]	Cardiovascular & PulmonaryDisease	retrospective cohort	China	frequent PVCs referred for catheter ablation	multifocal PVC; SHD; other cause of LVD	287	LVDD normalized after ablation
Liyun Zhang, 2016 [25]	CHINA MODERN MEDICINE	retrospective cohort	China	frequent PVCs referred for catheter ablation	SHD; other cause of LVD	96	LVEF normalized to ≥50%/ΔLVEF >15% after ablation
Baman TS, 2010 [15]	Heart Rhythm	retrospective cohort	America	frequent PVCs referred for catheter ablation	CAD	174	LVEF <50%
Koca H, 2020 [31]	Pace-Pacing and Clinical Electrophysiology	cohort	Turkey	symptomatic PVCs scheduled for RFCA	SHD/PVC recurrence	39	LVEF normalized to ≥50% after ablation
Krishnan B, 2017 [26]	JACC Clin Electrophysiol	cohort	America	PVC >10%/24 h underwent successful ablation	known cause of LVD	31	LVEF normalized to ≥50% after ablation
Maeda S, 2017 [34]	J Interv Card Electrophysiol	retrospective cohort	America	PVC-CMP underwent ablation	SHD	55	ΔLVEF >10 % after ablation
Mao J, 2021 [33]	Sci Rep	retrospective cohort	America	PVC-CMP undergone ablation	CAD/VHD/ARVC and cardiac sarcoidosis	19	ΔLVEF >10 % after ablation
Mountantonakis SE, 2011 [6]	Heart Rhythm	retrospective cohort	America	frequent PVCs (>5000/24 h) and LVEF <50% undergone ablation	/	69	ΔLVEF >5 % after ablation
Penela D, 2015 [35]	Heart Rhythm	prospective cohort	Spain; Argentina	frequent PVC (>4%) with criteria for PP-ICD implantation	survivors of SCD, sustained VT or syncope, previous ICD, or diagnosis of ARVC	66	removing the PP-ICD indication
Penela D, 2020 [36]	Europace	prospective cohort	Spain; Italy; Romania	frequent PVCs (>4%) and LVEF <50% undergone ablation	/	215	improvement of at least 5 absolute points in LVEF
Penela D, 2017 [37]	Heart Rhythm	prospective cohort	Spain; Argentina	frequent PVCs (>4%) and LVEF <50% undergone ablation	SHD	81	improvement of at least 5 absolute points in LVEF
Penela D, 2013 [38]	J Am Coll Cardiol	prospective cohort	Spain; Argentina; Netherlands	frequent PVCs (>4%) and LVEF <50%	/	80	improvement of at least 5 absolute points in LVEF
Deyell MW, 2012 [17]	Heart Rhythm	retrospective cohort	America	underwent successful ablation of PVCs (>10% PVCs/24) with LVEF <50%	a known cause for LVD or a history of sustained VT/appropriate ICD discharges or SCD	37	reversible (10% increase to a final LVEF of 50%)
Abdelhamid MA, 2018 [39]	Indian Heart J	cohort	Egypt	PVCCM (>10% PVCs/LVEF <50%) underwent ablation	sustained VT, CAD, atrial arrhythmias, NYHA III /IV, epicardial origin of PVCs	77	ΔLVEF >5 % after ablation
Wojdyła-Hordyńska A, 2017 [40]	Kardiol Pol	retrospective cohort	Germany; Poland	symptomatic frequent PVCs refractory to medical therapy, and with LVSD	sustained VT	109	/

Abbreviations: PVC, premature ventricular complex; RFCA, radiofrequency catheter ablation; SHD, structural heart disease; LVEF, left ventricular ejection fraction; LVDD, left ventricular diastolic diameter; CMRI, cardiac magnetic resonance imaging; CAD, coronary artery disease; VHD, valvular heart disease; ARVC, arrhythmogenic right ventricular cardiomyopathy; AF, atrial fibrillation; AFL, atrial flutter; VT, ventricular tachycardia; PP-ICD, primary prevention (PP) implantable cardioverter-defibrillator (ICD); PVC-CMP, premature ventricular complex (PVC) induced cardiomyopathy; SCD, sudden cardiac death; LVD, left ventricular dysfunction; HF, heart failure; OT, outflow tractc; RVOT, right ventricle outflow tract; DCM, dilated cardiomyopathy; IHD, ischemic heart disease; LVSD, left ventricular systolic dysfunction.

For PVC-CMP, 19 studies were designed as cohort studies (prospective/retrospective) with a follow-up time of 14–100 months and 6 studies were cross-sectional [2, 13, 16, 19, 27, 31]. Studies were located in Europe [3, 5, 20, 24, 28, 30, 31, 32], America [2, 3, 15, 16, 17, 18, 20, 21, 23, 24, 26, 32, 33], Asia [4, 13, 14, 19, 22, 25, 27] and Australia [29], and 10 developing countries, 10 developed countries and 5 multicenter studies [3, 5, 20, 24, 32]. Most of the 25 studies included symptomatic frequent PVC patients referred for catheter ablation other than 2 [2, 30]. A total of 7 did not emphasize the exclusion of the structural heart disease (SHD) [5, 16, 17, 24, 28, 30, 32]. The reduced left ventricular ejection fraction (LVEF) (<45–50% or ΔLVEF > –6%/–10%) was the endpoint of most studies except 2 (death or hospitalizations due to heart failure (HF) [22] and the enlargement of left ventricular [30]). 3 took the reversibility of LVEF/left ventricular end-diastolic dimension(LVEDD) after PVC ablation as the diagnostic criteria for PVC-CMP [18, 22, 25].

12 cohort studies from America [6, 17, 26, 33, 34, 37, 38], Europe [35, 36, 37, 38, 40], Egypt [39] and Turkey [31] explored the characteristics of patients who obtained complete or partial cardiac function restoration after PVC ablation after the 4–18 month follow-ups. 5 studies [6, 35, 36, 38, 39] incorporated coronary heart disease (CHD). The evaluation criteria for cardiac function reversibility was LVEF normalization [17, 26, 31, 40], LVEF increased by more than 5–10% or 5 absolute points [6, 33, 34, 36, 37, 38, 39], and removing the primary prevention (PP) implantable cardioverter-defibrillator (ICD) (PP-ICD) indication [35].

### 3.2 Factors Predicting PVC-CMP

**Fig. 2.**
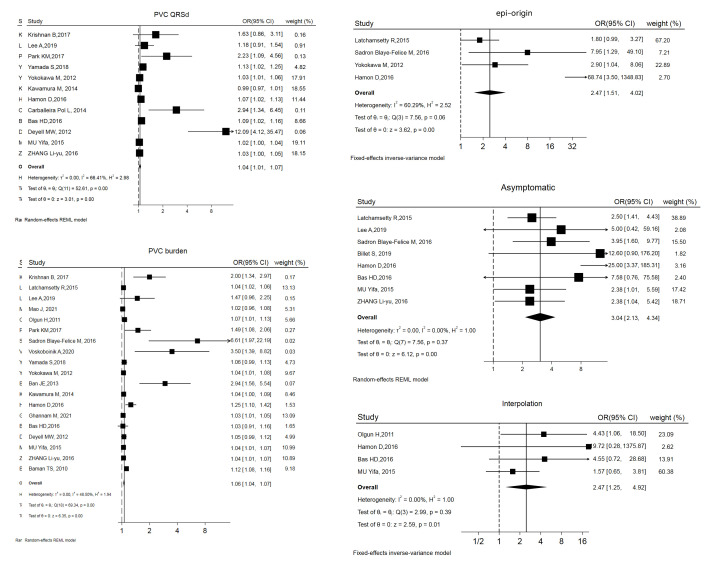
**Forest plot of odds ratios (OR) and 95% confidence intervals (CI) for the association between the 5 parameter and PVC-CMP**. PVC-CMP, premature ventricular complexes induced cardiomyopathy; PVC, premature ventricular complex; QRSd, QRS wave duration; epi, epicardial; REML, restricted maximum likelihood.

The variables and confounders are shown in Table 2 (Ref. [2, 3, 4, 5, 13, 14, 15, 16, 17, 18, 19, 20, 21, 22, 23, 24, 25, 26, 27, 28, 29, 30, 31, 32, 33]). 5 of them were included in more than 1 study, with multifactorial risk estimates (OR and 95% CI) reported in more than 50% of these studies. Merging the results revealed that 3 factors were significantly related to PVC-CMP: being asymptomatic (OR and 95% CI: 3.04 [2.13, 4.34]), interpolation (OR and 95% CI: 2.47 [1.25, 4.92]), and epicardial origin (epi-origin) (OR and 95% CI: 3.04 [2.13, 4.34]). Forest plots and funnel plots are displayed in Fig. 2 and **Supplementary Fig. 1**.

**Table 2.** **Summary of inclusion of variates in multi-variate models for association with PVC-CMP included in systematic review**.

First Author, Year	Age	Sex: male	Asymptomatic	PVC burden	PVC QRSd	PVC CI	LV-originated	Epi-originated	Single PVC	Ns-VT	RVOT	Interpolation	Inclusion	Others
Koca H, 2020 [31]	○	○	N/A	○	○	○	⚫	N/A	N/A	N/A	N/A	N/A	*p* < 0.05	Smoker; DM; **NT-proBNP**; AAD
Krishnan B, 2017 [26]	○	○	○	⚫	⚫	⚫	○	○	N/A	N/A	N/A	N/A	*p* < 0.2; PVC burden/QRSd	BMI; CAD; CHF; AAD
Latchamsetty R, 2015 [3]	⚫	⚫	⚫	⚫	N/A	N/A	N/A	⚫	⚫	N/A	⚫	N/A	*p* < 0.10	**CAD**; HTN; AAD
Lee A, 2019 [29]	○	⚫	⚫	⚫	⚫	○	N/A	○	N/A	N/A	⚫	N/A	*p* < 0.10 + reported	BMI; Scar; Smoker; CAD; AF; AAD
Mao J, 2021 [33]	⚫	⚫	N/A	⚫	N/A	N/A	⚫	N/A	⚫	N/A	⚫	N/A	fixed	**LV GLS**; Scar; AAD
Niwano S, 2009 [14]	○	○	N/A	○	N/A	N/A	N/A	N/A	N/A	N/A	N/A	N/A	/	**Basal LVEF**; AAD
Olgun H, 2011 [16]	○	○	N/A	⚫	N/A	N/A	N/A	N/A	N/A	N/A	N/A	⚫	*p* < 0.10	AAD
Park KM, 2017 [4]	○	⚫	N/A*	⚫	⚫	○	⚫	N/A	N/A	N/A	○	N/A	*p* < 0.10	HTN; AAD; Sinus QRSd
Parreira L, 2019 [30]	○	○	N/A	N/A	N/A	N/A	N/A	N/A	N/A	⚫	N/A	N/A	Fixed	CAD; HTN; DM; **PAC**; AAD
Sadron Blaye-Felice M, 2016 [5]	○	○	⚫	⚫	○	○	⚫	⚫	○	N/A	N/A	N/A	*p* < 0.05	AAD; **Sinus QRSd**
Voskoboinik A, 2020 [2]	○	○	○	⚫	○	⚫	○	N/A	○	⚫	N/A	N/A	*p* < 0.05	BMI; CAD; HTN; **Superior axis**; AAD
Yamada S, 2018 [27]	○	○	N/A	⚫	⚫	○	N/A	N/A	N/A	○	N/A	○	*p* < 0.05	**PDI**; AAD
Yokokawa M, 2012 [18]	○	⚫	N/A	⚫	⚫	N/A	○	⚫	N/A	N/A	○	N/A	*p* < 0.1	AAD
Ban JE, 2013 [19]	○	○	○	⚫	○	○	N/A	N/A	N/A	⚫	○	○	*p* < 0.05	**Retrograde P-wave**; AAD
Kanei Y, 2008 [13]	○	○	N/A	○	N/A	N/A	N/A	N/A	○	⚫	N/A*	N/A	fixed	Mean HR; AAD
Billet S, 2019 [28]	○	○	⚫	○	○	N/A	○	○	○	○	N/A	○	*p* < 0.1	AAD
Kawamura M, 2014 [21]	⚫	○	N/A	⚫	⚫	⚫	○	N/A	○	N/A	○	N/A	*p* < 0.1	**BMI**; AAD
Hamon D, 2016 [24]	○	⚫	⚫	⚫	⚫	⚫	⚫	⚫	N/A	N/A	N/A	⚫	*p* < 0.1	SHD; AAD; **Sinus QRSd**
Ghannam M, 2021 [32]	○	○	○	⚫	○	N/A	N/A	○	N/A	N/A	N/A	N/A	*p* < 0.05	Scar; AAD; Sinus QRSd
Carballeira Pol L, 2014 [20]	○	○	○	○	⚫	○	○	N/A	N/A	N/A	⚫	N/A	*p* < 0.1	AAD
Bas HD, 2016 [23]	○	⚫	⚫	⚫	⚫	N/A	N/A	N/A	⚫	N/A	N/A	⚫	*p* < 0.1	**Variation**; AAD
Deyell MW, 2012 [17]	○	○	○	⚫	⚫	N/A	○	N/A	○	○	○	N/A	*p* < 0.2	AF; AAD; Sinus QRSd
Yifan Mu, 2015 [22]	○	○	⚫	⚫	⚫	⚫	○	N/A	N/A*	○	N/A	⚫	fixed	Superior axis; **course of disease**
Liyun Zhang, 2016 [25]	○	○	⚫	⚫	⚫	⚫	N/A	N/A	N/A	N/A	N/A	N/A	fixed	**Mean HR; course of disease**
Baman TS, 2010 [15]	○	○	N/A	⚫	N/A	N/A	○	N/A	○	○	○	N/A	*p* < 0.1	**/**
Yield (%)	12.0	28.0	57.1	79.2	66.7	46.1	38.5	50.0	30.0	44.4	40.0	57.1	/	/

Abbreviations: PVC, premature ventricular complex; QRSd, QRS wave duration; CI, coupling interval; epi, epicardial; Ns-VT, non-sustained ventricular tachycardia; RVOT, right ventricle outflow tract; DM, diabetes mellitus; AAD, anti-arrhythmic drugs; BMI, body mass index; CAD, coronary artery disease; CHF, chronic heart failure; HTN, hypertension; AF, atrial fibrillation; LV GLS, left ventricular global longitudinal strain; LVEF, left ventricular ejection fraction; PAC, premature atrial complex; PDI, peak deflection index; HR, heart rate; SHD, structural heart disease. Annotations: ⚫ indicates that the variable included in the model and with the odds ratios (OR) provided; ○ indicates that the variable didn’t entered into the model or without OR (not independent factor). N/A indicates that the variable was not mentioned in the cohort; * indicates that the model could not include the variable because 100% of study individuals were in this category. Yield: the proportion of all the related studies which provided the OR of the variable ⚫ / ⚫ + ○. Bold means the independent factors.

### 3.3 Factors Associated with PVCs Exacerbated LVSD

**Fig. 3.**
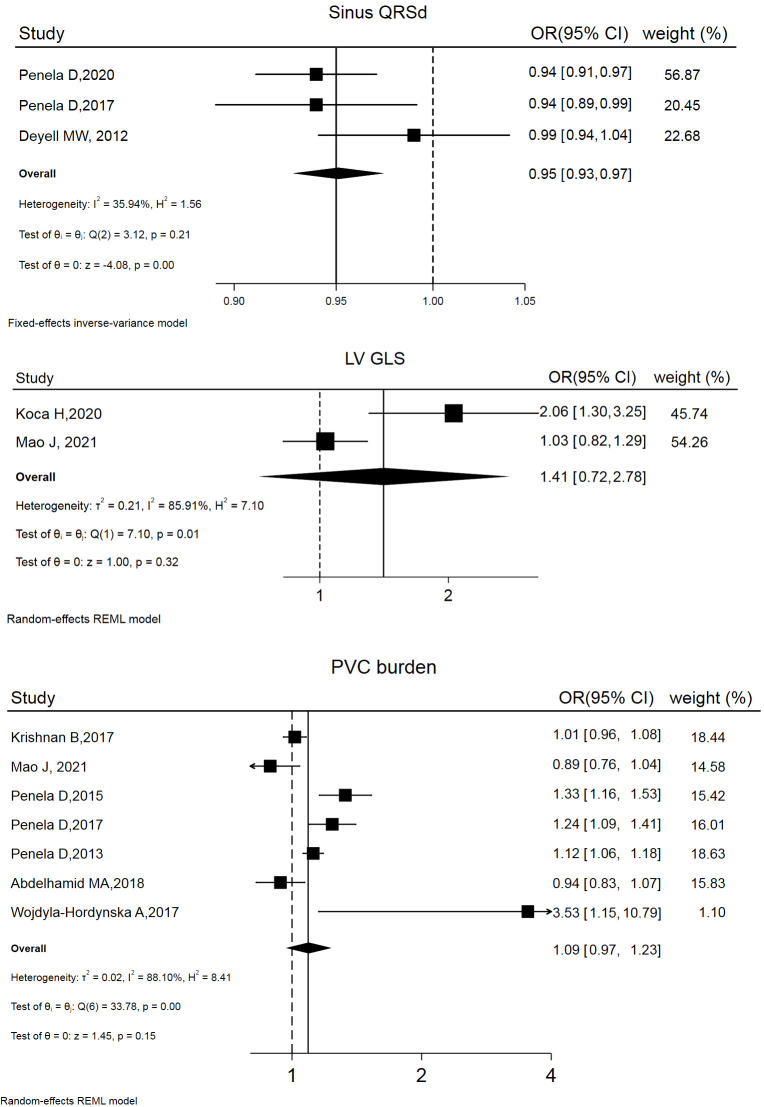
**Forest plot of odds ratios (OR) and 95% confidence intervals (CI) for the association between the 3 parameter and post-ablation LVSD reversibility**. LVSD, left ventricular systolic dysfunction; PVC, premature ventricular complex; QRSd, QRS wave duration; LV GLS, left ventricular global longitudinal strain; REML, restricted maximum likelihood.

12 studies addressed LVSD reversibility. Factors involved in models predicting post-ablation LVSD reversibility are summarized in Table 3 (Ref. [6, 17, 26, 31, 33, 34, 35, 36, 37, 38, 39, 40]). The risk estimates (OR and 95% CI) of 3 variables were combined, yielding the following results: left ventricular global longitudinal strain (LV GLS) (OR and 95% CI: 1.41 [0.72, 1.78]), PVC burden (OR and 95% CI: 1.09 [0.97, 1.23]), and sinus QRS wave duration (QRSd) (OR and 95% CI: 0.95 [0.93, 0.97]). Forest and funnel plots are displayed in Fig. 3 and **Supplemental Fig. 2**.

**Table 3.** **Summary of inclusion of variates in multi-variate models for association with post-ablation left ventricular systolic dysfunction reversibility included in systematic review**.

First Author, Year	Basal LVEF	PVC burden	PVC QRSd	Sinus QRSd	PVC CI	Single PVC	LV GLS	Inclusion criteria	Others
Koca H, 2020 [31]	⚫	N/A	N/A	N/A	○	○	⚫	*p* < 0.05	Age; NT-proBNP; LVDD
Krishnan B, 2017 [26]	○	⚫	⚫	N/A	⚫	○	N/A	*p* < 0.2; PVC burden/QRSd; basal LVEF	BMI; Asymptomatic; CHF; CAD; LV-originated; AAD
Maeda S, 2017 [34]	N/A	N/A	N/A	N/A	N/A	N/A	N/A	*p* < 0.1	**Sex (male)**; HTN; AAD
Mao J, 2021 [33]	⚫	⚫	N/A	N/A	N/A	⚫	⚫	Fixed	**Sex (male)**; **Age**; NT-proBNP; **LV-originated**; AAD
Mountantonakis SE, 2011 [6]	⚫	○	○	N/A	N/A	○	N/A	*p* < 0.1	Sex (male); Age;** Cardiomyopathy**; LV-originated
Penela D, 2015 [35]	N/A	⚫	○	N/A	N/A	N/A	N/A	*p* < 0.1	Sex (male); Age; CAD; LV-originated
Penela D, 2020 [36]	○	N/A	○	⚫	N/A	○	N/A	*p* < 0.1	Sex (male); Age; SHD; LV-originated
Penela D, 2017 [37]	○	⚫	○	⚫	N/A	○	N/A	*p* < 0.1	Sex (male); Age; NT-proBNP; Scar; LV-originated
Penela D, 2013 [38]	N/A	⚫	○	N/A	N/A	N/A	N/A	*p* < 0.1	Sex (male); Age; SHD; Scar; epi-origin; LV-originated
Deyell MW, 2012 [17]	⚫	○	⚫	⚫	○	○	○	*p* < 0.2	Age; Asymptomatic; CHF; AAD
Abdelhamid MA, 2018 [39]	○	⚫	N/A	N/A	N/A	N/A	N/A	/	Asymptomatic; SHD; epi-origin; LV-originated
Wojdyła-Hordyńska A, 2017 [40]	○	⚫	N/A	N/A	N/A	N/A	N/A	*p* < 0.3	Age; gender; SHD; site of origin
Yield (%)	44.4	77.8	28.6	100	33.0	14.3	66.7	/	/

Abbreviations: PVC, premature ventricular complex; LVEF, left ventricular ejection fraction; QRSd, QRS wave duration; CI, coupling interval; LV GLS, left ventricular global longitudinal strain; BMI, body mass index; LVDD, left ventricular diastolic diameter; CAD, coronary artery disease; CHF, chronic heart failure; AAD, anti-arrhythmic drugs; HTN, hypertension; AF, atrial fibrillation; LV, left ventricle; SHD, structural heart disease; epi, epicardial. Annotation**s**: ⚫ indicates that the variable included in the model and with the OR provided; ○ indicates that the variable didn’t entered into the model or without OR (not independent factor); N/A indicates that the variable was not mentioned in the cohort; Yield: the proportion of all the related studies which provided the OR of the variable ⚫ / ⚫ + ○. Bold means the independent factors.

## 4. Discussion

To our knowledge, this is the first meta-analysis to examine the independent risk factors of PVC-CMP and post-ablation LVSD reversibility. We identified 3 elements (asymptomatic PVCs, interpolation, and epicardial origin) that can independently predict PVC-CMP and 2 parameters (PVC burden and sinus QRS duration) significantly correlated with post-ablation LVSD reversibility.

PVC burden has been reported as a predictor for PVC-CMP [2, 5, 14], but the results have been controversial. Some studies suggested that at least a 10% PVC burden is required to induce PVC-cardiomyopathy [15, 41], while others disagreed [42, 43, 44]. Our meta-analysis showed that PVC burden had a weak relationship with PVC-CMP (OR and 95% CI: 1.06 [1.04, 1.07]), but significant publication bias was observed (Fig. 2 and Funnel plots in the **Supplemental Material**). Based on the literature review and our clinical experience, we tend to believe that PVC burden alone is not a strong risk factor for PVC-CMP. Del Carpio Munoz *et al*. [45] found that PVCs originating from the right ventricle (RV) with a lower daily burden than from the left ventricle (10% vs. 20%) resulted in LVEF impairment. This suggests that the location of PVCs may also play a role. Another reason for this discrepancy may be the misestimation of PVC burden by 24-hour Holter monitoring due to its day-to-day variability. Several studies have suggested that a PVC QRS duration >150 ms can predict PVC-CMP, but its role as an independent predictor remains controversial [18, 20, 24, 46]. The results of this meta-analysis showed that PVC QRS duration also does not play a key role in PVC-CMP (OR and 95% CI: 1.04 [1.01, 1.07]) (Fig. 2). In our opinion, PVC burden, location, and QRS duration collectively contribute to PVC-CMP by desynchronizing ventricular function. We found that PVCs originating in the epicardium had a stronger independent correlation (OR and 95% CI: 3.04 [2.13, 4.34]) (Fig. 2), which is consistent with previous studies [3, 5]. A possible explanation is that the reversed endocardial-epicardial depolarizing sequence is more harmful compared to pure electroexcitation delay (QRS prolongation) in terms of ventricular mechanical synchrony [47]. Regarding PVC location, some included studies identified it as an independent risk factor, such as right ventricular outflow tract (RVOT) origin (OR: 0.4–0.7) [3, 29], and left ventricle (LV) origin (OR: 1.2–4.5) [4, 31, 33]. However, quite a few studies [17, 18, 21, 28] reported negative results (without provided OR values) (Table 2). Due to such publication bias, we did not combine the OR values. Being asymptomatic was more consistently reported as a critical factor [3, 5, 24, 28, 29], with a combined OR of 3.04 (Fig. 2, Table 2). Asymptomatic status delays diagnosis and treatment, resulting in a longer duration of PVC exposure and making cardiomyopathy development more likely [18]. While the mechanism is not entirely clear, interpolated PVCs were also consistently found to convey a significant risk of developing cardiomyopathy [16, 22, 23, 24], with a combined OR of 2.47 (Fig. 2, Table 2). 4 studies [2, 13, 19, 30] reported that the presence of non-sustained ventricular tachycardia (NSVT) was independently related to PVC-CMP, but the risk estimates were not combined due to publication bias (Table 2). It is unclear whether NSVT promotes cardiomyopathy progression through the “tachycardia-induced cardiomyopathy” mechanism or if it is merely an early sign of cardiomyopathy.

Not all patients diagnosed with PVC-CMP will benefit from PVC ablation in terms of improving cardiac function [17, 26, 34]. In cases of SHD, PVC ablation is generally considered helpful for cardiac reverse remodeling [6, 7, 8]. It appears that determining the extent and timing of PVC’s causal role in cardiomyopathy is complex, possibly dynamic. A previous meta-regression [48] and a recent systematic review [49] indicated that PVC burden, LVEF, QRS duration, the absence of underlying cardiomyopathy, younger age, variability in the frequency of PVCs, and lower left ventricular end-diastolic diameter (LVEDD), but not the origin of PVCs, were predictive of post-ablation LVEF improvement. According to this meta-analysis, 2 factors independently correlated with post-ablation LVEF improvement: a greater PVC burden and a narrower sinus QRS duration. Basal LVEF and concurrent SHD do not appear to be crucial. As previously mentioned, the extent to which ventricular asynchrony caused by PVCs worsens cardiac function is a key consideration in determining the benefit of PVC elimination (ablation) for cardiac resynchronization therapy. It is logical to assume that a higher PVC burden results in a greater degree of asynchrony, and a narrower sinus QRS duration indicates better synchronization achieved through PVC ablation.

## 5. Strengths and Limitations 

The principal strength of this study lies in the systematic literature search conducted by independent reviewers who searched comprehensive medical literature databases. We extracted and combined risk estimates (OR and 95% CI) obtained through multifactorial analysis, taking publication bias into consideration. Additionally, all included studies exhibited relatively high quality.

However, this study has several limitations. First, there is considerable heterogeneity among the included studies in terms of design, inclusion criteria, and evaluation criteria. Some studies may share partial cohorts [35, 36, 37, 38]. Second, a selection bias is likely present because most studies enrolled patients referred for ablation due to frequent, symptomatic, and medical refractory PVCs. Third, despite contacting corresponding authors via email, the risk estimates (OR and 95% CI) for some variables included in the multifactorial analysis model are unavailable.

## 6. Conclusions

In conclusion, current studies show considerable inconsistency regarding the prediction of PVC-CMP and post-ablation LVSD reversibility. The relatively consistent independent risk factors for PVC-CMP and post-ablation LVSD reversibility are asymptomatic status, interpolation, epicardial origin, PVC burden, and sinus QRS duration, respectively.

## Availability of Data and Materials

The datasets used and/or analyzed during the current study are available from the corresponding author on reasonable request.

## Supplementary Material

Supplementary material associated with this article can be found, in the online version, at https://doi.org/10.31083/j.rcm2509327.
