# Supplementary material for: Risk Factors for PVC Induced Cardiomyopathy and Post-Ablation Left Ventricular Systolic Dysfunction Reversibility: A Systematic Review and Meta-Analysis of Observational Studies
Source: Rev Cardiovasc Med. 2024 Sep 11;25(9):327. doi: 10.31083/j.rcm2509327 (PMC11440414; doi:10.31083/j.rcm2509327)
Supplement: Supplementary file 1 [file 2153-8174-25-9-327-s1.zip › Supplementary material 1 The full literature search strategy.docx]

Supplement 1: Search Strategy

| Patient population | | Study |
| --- | --- | --- |
| "ventricular contraction*" | "Cardiomyopathies"[Mh] | "Cross-Sectional Studies"[Mh] |
| "Ventricular Ectopic" | "Heart Failure"[Mh] | "Case-Control Studies" |
| "ventricular extrasystole*" | "ventricular dysfunction, left"[Mh] | "Cohort Studies"[Mh] |
| "Ventricular Premature Complexes"[Mh] | "left ventricular ejection fraction" | "Observational Studies as Topic"[Mh] |

**Pubmed:**

PVC:

"Ventricular Premature Complexes"[MeSH Terms] OR "ventricular premature complexe*"[Title/Abstract] OR "Ventricular Complex*"[Title/Abstract] OR "ventricular contraction*"[Title/Abstract] OR "ventricular extrasystole*"[Title/Abstract] OR "ventricular beat*"[Title/Abstract] OR "Ventricular Premature"[Title/Abstract] OR "Ventricular Ectopic"[Title/Abstract]

LVSD:

"Cardiomyopathies"[MeSH Terms] OR "Heart Failure"[MeSH Terms] OR "cardiomyopathy"[Title/Abstract] OR "ventricular dysfunction, left"[MeSH Terms] OR "Ventricular Dysfunction"[Title/Abstract] OR "Heart Failure"[Title/Abstract] OR "left ventricular ejection fraction"[Title/Abstract]

Study:

"Cross-Sectional Studies"[MeSH Terms] OR "Case-Control Studies"[MeSH Terms] OR "Cohort Studies"[MeSH Terms] OR "Observational Study"[Publication Type] OR "Observational Studies as Topic"[MeSH Terms] OR "Case-Control Studies"[Title/Abstract] OR "cohort"[Title/Abstract] OR "prospective"[Title/Abstract] OR "retrospective"[Title/Abstract] OR "control group*"[Title/Abstract] OR "independent predictor"[Title/Abstract] OR "followed-up"[Title/Abstract] OR "Follow-Up Studies"[MeSH Terms]

**Web of Science:**

PVC:

TS=("Ventricular Premature Complexes") OR TS=("ventricular premature complex*") OR TS=("ventricular extrasystole*") OR TS=("ventricular contraction*") OR TS=("ventricular premature beat") OR TS=("ventricular complex*")

LVSD:

TS=(Cardiomyopathies) OR TS=("heart failure") OR TS=(cardiomyopathy) OR TS=("ventricular dysfunction") OR TS=(cardiomegaly) OR TS=("cardiac dilatation") OR TS=("left ventricular ejection fraction")

Study:

TS=("Cross-Sectional") OR TS=("Case-Control") OR TS=(Cohort ) OR TS=(Observational) OR TS=(prospective) OR TS=(case) OR TS=(control) OR TS=(retrospective) OR TS=("independent predictor") OR TS=("followed-up")

**Embase:**

PVC:

'ventricular premature comple*':ab,ti AND [article]/lim AND [humans]/lim AND [english]/lim AND [clinical study]/lim AND [embase]/lim OR ('ventricular complex*':ab,ti AND ([article]/lim OR [article in press]/lim) AND [humans]/lim AND [english]/lim AND [clinical study]/lim AND [embase]/lim) OR ('ventricular contraction*':ab,ti AND ([article]/lim OR [article in press]/lim) AND [humans]/lim AND [english]/lim AND [clinical study]/lim AND [embase]/lim) OR ('ventricular extrasystole*':ab,ti AND ([article]/lim OR [article in press]/lim) AND [humans]/lim AND [english]/lim AND [clinical study]/lim AND [embase]/lim) OR ('ventricular ectopic':ab,ti AND ([article]/lim OR [article in press]/lim) AND [humans]/lim AND [clinical study]/lim AND [embase]/lim) OR ('ventricular premature':ab,ti AND ([article]/lim OR [article in press]/lim) AND [humans]/lim AND [english]/lim AND [clinical study]/lim AND [embase]/lim) OR ('ventricular beat*':ab,ti AND ([article]/lim OR [article in press]/lim) AND [humans]/lim AND [english]/lim AND [clinical study]/lim AND [embase]/lim) OR (('heart ventricle extrasystole'/exp OR 'heart ventricle extrasystole') AND ([article]/lim OR [article in press]/lim) AND [humans]/lim AND [english]/lim AND [clinical study]/lim AND [embase]/lim)

LVSD:

('cardiomyopathy'/exp OR 'cardiomyopathy') AND ([article]/lim OR [article in press]/lim) AND [humans]/lim AND [english]/lim AND [clinical study]/lim AND [embase]/lim OR (('heart failure'/exp OR 'heart failure') AND ([article]/lim OR [article in press]/lim) AND [humans]/lim AND [english]/lim AND [clinical study]/lim AND [embase]/lim) OR (('heart left ventricle failure'/exp OR 'heart left ventricle failure') AND ([article]/lim OR [article in press]/lim) AND [humans]/lim AND [english]/lim AND [clinical study]/lim AND [embase]/lim) OR (('cardiomegaly'/exp OR 'cardiomegaly') AND ([article]/lim OR [article in press]/lim) AND [humans]/lim AND [english]/lim AND [clinical study]/lim AND [embase]/lim) OR (('heart dilatation'/exp OR 'heart dilatation') AND ([article]/lim OR [article in press]/lim) AND [humans]/lim AND [english]/lim AND [clinical study]/lim AND [embase]/lim)

Study:

('cross-sectional study'/exp OR 'cross-sectional study') AND ([article]/lim OR [article in press]/lim) AND [humans]/lim AND [english]/lim AND [clinical study]/lim AND [embase]/lim OR (('case control study'/exp OR 'case control study') AND ([article]/lim OR [article in press]/lim) AND [humans]/lim AND [english]/lim AND [clinical study]/lim AND [embase]/lim) OR (('cohort analysis'/exp OR 'cohort analysis') AND ([article]/lim OR [article in press]/lim) AND [humans]/lim AND [english]/lim AND [clinical study]/lim AND [embase]/lim) OR (('observational study'/exp OR 'observational study') AND ([article]/lim OR [article in press]/lim) AND [humans]/lim AND [english]/lim AND [clinical study]/lim AND [embase]/lim)
