# Supplementary material for: Risk Factors for PVC Induced Cardiomyopathy and Post-Ablation Left Ventricular Systolic Dysfunction Reversibility: A Systematic Review and Meta-Analysis of Observational Studies
Source: Rev Cardiovasc Med. 2024 Sep 11;25(9):327. doi: 10.31083/j.rcm2509327 (PMC11440414; doi:10.31083/j.rcm2509327)
Supplement: Supplementary file 1 [file 2153-8174-25-9-327-s1.zip › Supplementary material 3 Quality assessment of the eligible studies.docx]

**Table 1. The Newcastle-Ottawa Scale for quality assessment of cohort studies**

|  | Selection | | | | Comparability | Outcome | | |  |
| --- | --- | --- | --- | --- | --- | --- | --- | --- | --- |
| First Author, Year | Representativeness of the exposed cohort | Selection of the non-exposed cohort | Ascertainment of exposure | Demonstration that outcome of interest was not present at start of study | Comparability of cohorts on the basis of the design or analysis | Assessment of outcome | Was follow-up long enough for outcomes to occur | Adequacy of follow-up for cohorts | Total score |
| Krishnan B, 2017 | B* | A* | A* | A* | A*B* | A* | A* | C | 8 |
| Latchamsetty R,2015 | B* | A* | A* | A* | A*B* | A* | A* | A* | 9 |
| Lee A,2019 | B* | A* | A* | A* | A*B* | A* | A* | A* | 9 |
| Mao J, 2021 | B* | A* | A* | A* | A*B* | A* | A* | B* | 9 |
| Niwano S,2009 | B* | A* | A* | A* | A*B* | A* | A* | A* | 9 |
| Park KM,2017 | B* | A* | A* | A* | A*B* | A* | B | D | 7 |
| Parreira L,2019 | B* | A* | A* | A* | A*B* | A* | A* | A* | 9 |
| Sadron Blaye-Felice M, 2016 | B* | A* | A* | A* | A*B* | A* | A* | A* | 9 |
| Yokokawa M, 2012 | B* | A* | A* | A* | A*B* | A* | A* | A* | 9 |
| Billet S, 2019 | B* | A* | A* | A* | A*B* | A* | A* | A* | 9 |
| Kawamura M, 2014 | B* | A* | A* | A* | A*B* | A* | A* | A* | 9 |
| Hamon D,2016 | B* | A* | A* | A* | A*B* | A* | A* | A* | 9 |
| Ghannam M, 2021 | B* | A* | A* | A* | A*B* | A* | A* | A* | 9 |
| Carballeira Pol L, 2014 | B* | A* | A* | A* | A*B* | A* | A* | A* | 9 |
| Bas HD,2016 | B* | A* | A* | A* | A*B* | A* | A* | A* | 9 |
| Deyell MW, 2012 | B* | A* | A* | A* | A*B* | A* | A* | B* | 9 |
| MU Yifa, 2015 | B* | A* | A* | A* | A*B* | A* | A* | A* | 9 |
| ZHANG Li-yu, 2016 | B* | A* | A* | A* | A*B* | A* | A* | A* | 9 |
| Krishnan B,2017 | B* | A* | A* | A* | A*B* | A* | A* | B* | 9 |
| Maeda S,2017 | B* | A* | A* | A* | A*B* | A* | A* | A* | 9 |
| Mao J, 2021 | B* | A* | A* | A* | A*B* | A* | A* | B* | 9 |
| Mountantonakis SE,2011 | B* | A* | A* | A* | A*B* | A* | A* | A* | 9 |
| Penela D,2015 | B* | A* | A* | A* | A*B* | A* | A* | A* | 9 |
| Penela D,2020 | B* | A* | A* | A* | A*B* | A* | A* | A* | 9 |
| Penela D,2017 | B* | A* | A* | A* | A*B* | A* | A* | A* | 9 |
| Penela D,2013 | B* | A* | A* | A* | A*B* | A* | A* | A* | 9 |
| Abdelhamid MA,2018 | B* | A* | A* | A* | A*B* | A* | A* | A* | 9 |
| Wojdyla-Hordynska, A.2017 | B* | A* | A* | A* | A*B* | A* | A* | A* | 9 |

Selection: (1) A*: truly representative of the average adult population in the community; B*: somewhat representative of the adult population in the community; C: selected group; D: no description of the derivation of the cohort. (2) A*: Selection of the non-intervention cohort drawn from the same community as the intervention cohort; B: drawn from a different source; C: no description of the derivation of the non-intervention cohort. (3) A*: secure record; B*: structured interview; C: written self-report; D: no description. (4) A*: yes; B: no. Comparability: (A maximum of two stars can be given for Comparability). (1) A*: study controls for age, cardiovascular disease; B*: study controls for sex, diabetes, treatment, symptoms. Outcome: (1) A*: independent blind assessment; B*: record linkage; C: self-report; D: no description. (2) A*: yes ; B: no (3) A*: complete follow-up; all subjects were accounted for; B*: Subjects lost to follow-up were unlikely to introduce bias because small numbers were lost; >_80% had follow up, or description was provided of those lost; C: follow-up rate <80%, and there was no description of those lost; D: no statement.

**Table 2. Quality assessment of cross-sectional studies**

| **First Author,Year** | **1) Define the source of information (survey, record review)** | **2) List inclusion and exclusion criteria for exposed and unexposed subjects (cases and controls) or refer to previous publications** | **3) Indicate time period used for identifying patients** | **4) Indicate whether or not subjects were consecutive if not population-based** | **5) Indicate if evaluators of subjective components of study were masked to other aspects of the status of the participants** | **6) Describe any assessments undertaken for quality assurance purposes** | **7) Explain any patient exclusions from analysis** | **8) Describe how confounding was assessed and/or controlled.** | **9) If applicable, explain how missing data were handled in the analysis** | **10) Summarize patient response rates and completeness of data collection** | **11) Clarify what follow-up, if any, was expected and the percentage of patients for which incomplete data or follow-up was obtained** | **Total quality score** |
| --- | --- | --- | --- | --- | --- | --- | --- | --- | --- | --- | --- | --- |
| Koca H,2020 | 1 | 1 | 1 | 0 | 0 | 1 | 0 | 1 | 0 | 0 | 0 | 5 |
| Olgun H,2011 | 1 | 0 | 0 | 0 | 0 | 1 | 0 | 1 | 0 | 0 | 1 | 4 |
| Voskoboinik A,2020 | 1 | 0 | 1 | 0 | 0 | 1 | 0 | 1 | 0 | 0 | 0 | 4 |
| Yamada S,2018 | 1 | 1 | 1 | 0 | 0 | 1 | 0 | 1 | 0 | 0 | 1 | 6 |
| Ban JE,2013 | 1 | 1 | 0 | 0 | 0 | 1 | 0 | 1 | 0 | 1 | 1 | 6 |
| Kanei Y,2008 | 1 | 1 | 1 | 0 | 0 | 1 | 1 | 1 | 0 | 0 | 0 | 6 |
| Baman, T. S,2010 | 1 | 1 | 0 | 0 | 0 | 1 | 0 | 1 | 0 | 0 | 1 | 5 |

The study quality was assessed according to the 11 items recommended by the Agency for Healthcare Research and Quality (AHRQ) for cross-sectional studies. 1 point if the item was contemplated in the study, 0 point if the item was not, and unable to determine. 1 = “Yes”, 0 = “No”, “Unable to determine”, or “Not applicable”
