# Supplementary material for: Risk Factors for PVC Induced Cardiomyopathy and Post-Ablation Left Ventricular Systolic Dysfunction Reversibility: A Systematic Review and Meta-Analysis of Observational Studies
Source: Rev Cardiovasc Med. 2024 Sep 11;25(9):327. doi: 10.31083/j.rcm2509327 (PMC11440414; doi:10.31083/j.rcm2509327)
Supplement: Supplementary file 1 [file 2153-8174-25-9-327-s1.zip › Supplementary material.docx]

Supplementary material 1: Search Strategy

| Patient population | | Study |
| --- | --- | --- |
| "ventricular contraction*" | "Cardiomyopathies"[Mh] | "Cross-Sectional Studies"[Mh] |
| "Ventricular Ectopic" | "Heart Failure"[Mh] | "Case-Control Studies" |
| "ventricular extrasystole*" | "ventricular dysfunction, left"[Mh] | "Cohort Studies"[Mh] |
| "Ventricular Premature Complexes"[Mh] | "left ventricular ejection fraction" | "Observational Studies as Topic"[Mh] |

**Pubmed:**

PVC:

"Ventricular Premature Complexes"[MeSH Terms] OR "ventricular premature complexe*"[Title/Abstract] OR "Ventricular Complex*"[Title/Abstract] OR "ventricular contraction*"[Title/Abstract] OR "ventricular extrasystole*"[Title/Abstract] OR "ventricular beat*"[Title/Abstract] OR "Ventricular Premature"[Title/Abstract] OR "Ventricular Ectopic"[Title/Abstract]

LVSD:

"Cardiomyopathies"[MeSH Terms] OR "Heart Failure"[MeSH Terms] OR "cardiomyopathy"[Title/Abstract] OR "ventricular dysfunction, left"[MeSH Terms] OR "Ventricular Dysfunction"[Title/Abstract] OR "Heart Failure"[Title/Abstract] OR "left ventricular ejection fraction"[Title/Abstract]

Study:

"Cross-Sectional Studies"[MeSH Terms] OR "Case-Control Studies"[MeSH Terms] OR "Cohort Studies"[MeSH Terms] OR "Observational Study"[Publication Type] OR "Observational Studies as Topic"[MeSH Terms] OR "Case-Control Studies"[Title/Abstract] OR "cohort"[Title/Abstract] OR "prospective"[Title/Abstract] OR "retrospective"[Title/Abstract] OR "control group*"[Title/Abstract] OR "independent predictor"[Title/Abstract] OR "followed-up"[Title/Abstract] OR "Follow-Up Studies"[MeSH Terms]

**Web of Science:**

PVC:

TS=("Ventricular Premature Complexes") OR TS=("ventricular premature complex*") OR TS=("ventricular extrasystole*") OR TS=("ventricular contraction*") OR TS=("ventricular premature beat") OR TS=("ventricular complex*")

LVSD:

TS=(Cardiomyopathies) OR TS=("heart failure") OR TS=(cardiomyopathy) OR TS=("ventricular dysfunction") OR TS=(cardiomegaly) OR TS=("cardiac dilatation") OR TS=("left ventricular ejection fraction")

Study:

TS=("Cross-Sectional") OR TS=("Case-Control") OR TS=(Cohort ) OR TS=(Observational) OR TS=(prospective) OR TS=(case) OR TS=(control) OR TS=(retrospective) OR TS=("independent predictor") OR TS=("followed-up")

**Embase:**

PVC:

'ventricular premature comple*':ab,ti AND [article]/lim AND [humans]/lim AND [english]/lim AND [clinical study]/lim AND [embase]/lim OR ('ventricular complex*':ab,ti AND ([article]/lim OR [article in press]/lim) AND [humans]/lim AND [english]/lim AND [clinical study]/lim AND [embase]/lim) OR ('ventricular contraction*':ab,ti AND ([article]/lim OR [article in press]/lim) AND [humans]/lim AND [english]/lim AND [clinical study]/lim AND [embase]/lim) OR ('ventricular extrasystole*':ab,ti AND ([article]/lim OR [article in press]/lim) AND [humans]/lim AND [english]/lim AND [clinical study]/lim AND [embase]/lim) OR ('ventricular ectopic':ab,ti AND ([article]/lim OR [article in press]/lim) AND [humans]/lim AND [clinical study]/lim AND [embase]/lim) OR ('ventricular premature':ab,ti AND ([article]/lim OR [article in press]/lim) AND [humans]/lim AND [english]/lim AND [clinical study]/lim AND [embase]/lim) OR ('ventricular beat*':ab,ti AND ([article]/lim OR [article in press]/lim) AND [humans]/lim AND [english]/lim AND [clinical study]/lim AND [embase]/lim) OR (('heart ventricle extrasystole'/exp OR 'heart ventricle extrasystole') AND ([article]/lim OR [article in press]/lim) AND [humans]/lim AND [english]/lim AND [clinical study]/lim AND [embase]/lim)

LVSD:

('cardiomyopathy'/exp OR 'cardiomyopathy') AND ([article]/lim OR [article in press]/lim) AND [humans]/lim AND [english]/lim AND [clinical study]/lim AND [embase]/lim OR (('heart failure'/exp OR 'heart failure') AND ([article]/lim OR [article in press]/lim) AND [humans]/lim AND [english]/lim AND [clinical study]/lim AND [embase]/lim) OR (('heart left ventricle failure'/exp OR 'heart left ventricle failure') AND ([article]/lim OR [article in press]/lim) AND [humans]/lim AND [english]/lim AND [clinical study]/lim AND [embase]/lim) OR (('cardiomegaly'/exp OR 'cardiomegaly') AND ([article]/lim OR [article in press]/lim) AND [humans]/lim AND [english]/lim AND [clinical study]/lim AND [embase]/lim) OR (('heart dilatation'/exp OR 'heart dilatation') AND ([article]/lim OR [article in press]/lim) AND [humans]/lim AND [english]/lim AND [clinical study]/lim AND [embase]/lim)

Study:

('cross-sectional study'/exp OR 'cross-sectional study') AND ([article]/lim OR [article in press]/lim) AND [humans]/lim AND [english]/lim AND [clinical study]/lim AND [embase]/lim OR (('case control study'/exp OR 'case control study') AND ([article]/lim OR [article in press]/lim) AND [humans]/lim AND [english]/lim AND [clinical study]/lim AND [embase]/lim) OR (('cohort analysis'/exp OR 'cohort analysis') AND ([article]/lim OR [article in press]/lim) AND [humans]/lim AND [english]/lim AND [clinical study]/lim AND [embase]/lim) OR (('observational study'/exp OR 'observational study') AND ([article]/lim OR [article in press]/lim) AND [humans]/lim AND [english]/lim AND [clinical study]/lim AND [embase]/lim)

Supplementary material 2 Quality assessment of the eligible studies

Supplementary Table 1. The Newcastle-Ottawa Scale for quality assessment of cohort studies.

|  | Selection | | | | Comparability | Outcome | | |  |
| --- | --- | --- | --- | --- | --- | --- | --- | --- | --- |
| First Author, Year | Representativeness of the exposed cohort | Selection of the non-exposed cohort | Ascertainment of exposure | Demonstration that outcome of interest was not present at start of study | Comparability of cohorts on the basis of the design or analysis | Assessment of outcome | Was follow-up long enough for outcomes to occur | Adequacy of follow-up for cohorts | Total score |
| Krishnan B, 2017 | B* | A* | A* | A* | A*B* | A* | A* | C | 8 |
| Latchamsetty R,2015 | B* | A* | A* | A* | A*B* | A* | A* | A* | 9 |
| Lee A,2019 | B* | A* | A* | A* | A*B* | A* | A* | A* | 9 |
| Mao J, 2021 | B* | A* | A* | A* | A*B* | A* | A* | B* | 9 |
| Niwano S,2009 | B* | A* | A* | A* | A*B* | A* | A* | A* | 9 |
| Park KM,2017 | B* | A* | A* | A* | A*B* | A* | B | D | 7 |
| Parreira L,2019 | B* | A* | A* | A* | A*B* | A* | A* | A* | 9 |
| Sadron Blaye-Felice M, 2016 | B* | A* | A* | A* | A*B* | A* | A* | A* | 9 |
| Yokokawa M, 2012 | B* | A* | A* | A* | A*B* | A* | A* | A* | 9 |
| Billet S, 2019 | B* | A* | A* | A* | A*B* | A* | A* | A* | 9 |
| Kawamura M, 2014 | B* | A* | A* | A* | A*B* | A* | A* | A* | 9 |
| Hamon D,2016 | B* | A* | A* | A* | A*B* | A* | A* | A* | 9 |
| Ghannam M, 2021 | B* | A* | A* | A* | A*B* | A* | A* | A* | 9 |
| Carballeira Pol L, 2014 | B* | A* | A* | A* | A*B* | A* | A* | A* | 9 |
| Bas HD,2016 | B* | A* | A* | A* | A*B* | A* | A* | A* | 9 |
| Deyell MW, 2012 | B* | A* | A* | A* | A*B* | A* | A* | B* | 9 |
| MU Yifa, 2015 | B* | A* | A* | A* | A*B* | A* | A* | A* | 9 |
| ZHANG Li-yu, 2016 | B* | A* | A* | A* | A*B* | A* | A* | A* | 9 |
| Krishnan B,2017 | B* | A* | A* | A* | A*B* | A* | A* | B* | 9 |
| Maeda S,2017 | B* | A* | A* | A* | A*B* | A* | A* | A* | 9 |
| Mao J, 2021 | B* | A* | A* | A* | A*B* | A* | A* | B* | 9 |
| Mountantonakis SE,2011 | B* | A* | A* | A* | A*B* | A* | A* | A* | 9 |
| Penela D,2015 | B* | A* | A* | A* | A*B* | A* | A* | A* | 9 |
| Penela D,2020 | B* | A* | A* | A* | A*B* | A* | A* | A* | 9 |
| Penela D,2017 | B* | A* | A* | A* | A*B* | A* | A* | A* | 9 |
| Penela D,2013 | B* | A* | A* | A* | A*B* | A* | A* | A* | 9 |
| Abdelhamid MA,2018 | B* | A* | A* | A* | A*B* | A* | A* | A* | 9 |
| Wojdyla-Hordynska, A.2017 | B* | A* | A* | A* | A*B* | A* | A* | A* | 9 |

Selection: (1) A*: truly representative of the average adult population in the community; B*: somewhat representative of the adult population in the community; C: selected group; D: no description of the derivation of the cohort. (2) A*: Selection of the non-intervention cohort drawn from the same community as the intervention cohort; B: drawn from a different source; C: no description of the derivation of the non-intervention cohort. (3) A*: secure record; B*: structured interview; C: written self-report; D: no description. (4) A*: yes; B: no. Comparability: (A maximum of two stars can be given for Comparability). (1) A*: study controls for age, cardiovascular disease; B*: study controls for sex, diabetes, treatment, symptoms. Outcome: (1) A*: independent blind assessment; B*: record linkage; C: self-report; D: no description. (2) A*: yes ; B: no (3) A*: complete follow-up; all subjects were accounted for; B*: Subjects lost to follow-up were unlikely to introduce bias because small numbers were lost; >_80% had follow up, or description was provided of those lost; C: follow-up rate <80%, and there was no description of those lost; D: no statement.

Supplementary Table 2. Quality assessment of cross-sectional studies

| **First Author,Year** | **1) Define the source of information (survey, record review)** | **2) List inclusion and exclusion criteria for exposed and unexposed subjects (cases and controls) or refer to previous publications** | **3) Indicate time period used for identifying patients** | **4) Indicate whether or not subjects were consecutive if not population-based** | **5) Indicate if evaluators of subjective components of study were masked to other aspects of the status of the participants** | **6) Describe any assessments undertaken for quality assurance purposes** | **7) Explain any patient exclusions from analysis** | **8) Describe how confounding was assessed and/or controlled.** | **9) If applicable, explain how missing data were handled in the analysis** | **10) Summarize patient response rates and completeness of data collection** | **11) Clarify what follow-up, if any, was expected and the percentage of patients for which incomplete data or follow-up was obtained** | **Total quality score** |
| --- | --- | --- | --- | --- | --- | --- | --- | --- | --- | --- | --- | --- |
| Koca H,2020 | 1 | 1 | 1 | 0 | 0 | 1 | 0 | 1 | 0 | 0 | 0 | 5 |
| Olgun H,2011 | 1 | 0 | 0 | 0 | 0 | 1 | 0 | 1 | 0 | 0 | 1 | 4 |
| Voskoboinik A,2020 | 1 | 0 | 1 | 0 | 0 | 1 | 0 | 1 | 0 | 0 | 0 | 4 |
| Yamada S,2018 | 1 | 1 | 1 | 0 | 0 | 1 | 0 | 1 | 0 | 0 | 1 | 6 |
| Ban JE,2013 | 1 | 1 | 0 | 0 | 0 | 1 | 0 | 1 | 0 | 1 | 1 | 6 |
| Kanei Y,2008 | 1 | 1 | 1 | 0 | 0 | 1 | 1 | 1 | 0 | 0 | 0 | 6 |
| Baman, T. S,2010 | 1 | 1 | 0 | 0 | 0 | 1 | 0 | 1 | 0 | 0 | 1 | 5 |

The study quality was assessed according to the 11 items recommended by the Agency for Healthcare Research and Quality (AHRQ) for cross-sectional studies. 1 point if the item was contemplated in the study, 0 point if the item was not, and unable to determine. 1 = “Yes”, 0 = “No”, “Unable to determine”, or “Not applicable”

Supplementary material 3 The funnel plots


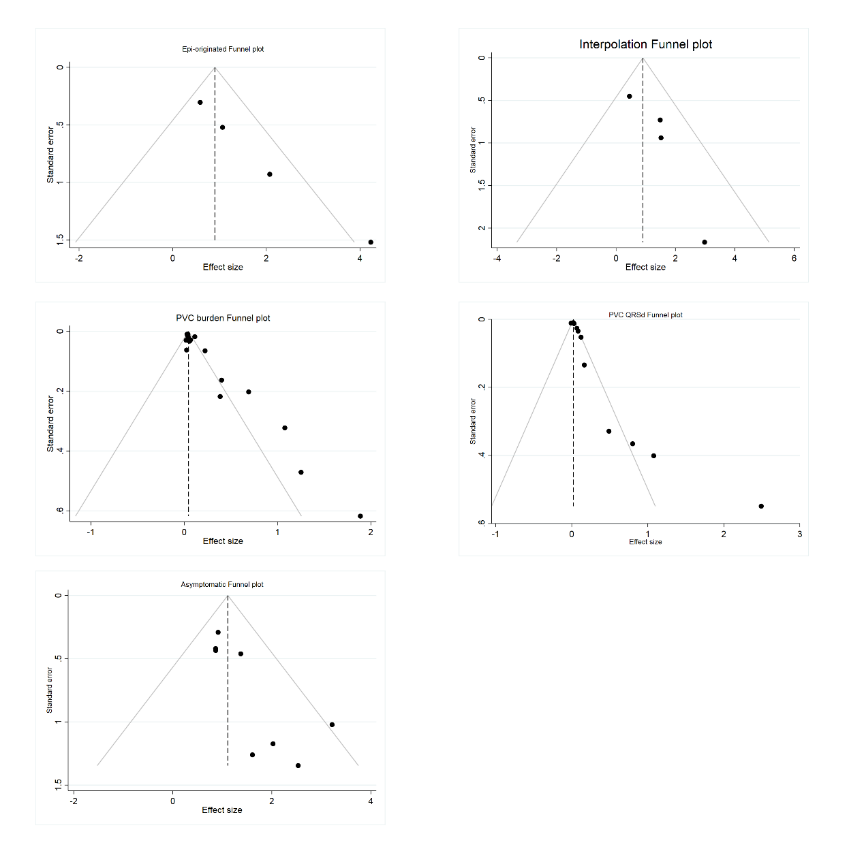


**Supplementary Fig. 1.** **The funnel plots of factors predicting PVC-CMP**


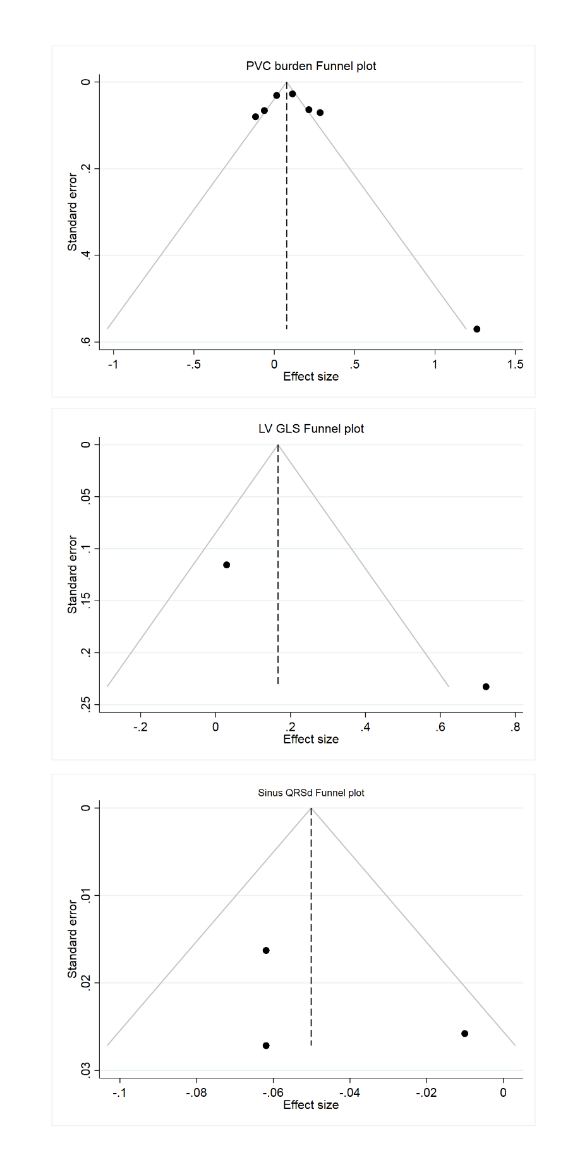


**Supplementary Fig. 2**. **The funnel plots of factors associated with PVCs exacerbated LVSD**
