# Supplementary figures and images for: Risk Factors for PVC Induced Cardiomyopathy and Post-Ablation Left Ventricular Systolic Dysfunction Reversibility: A Systematic Review and Meta-Analysis of Observational Studies
Source: Rev Cardiovasc Med. 2024 Sep 11;25(9):327. doi: 10.31083/j.rcm2509327 (PMC11440414; doi:10.31083/j.rcm2509327)

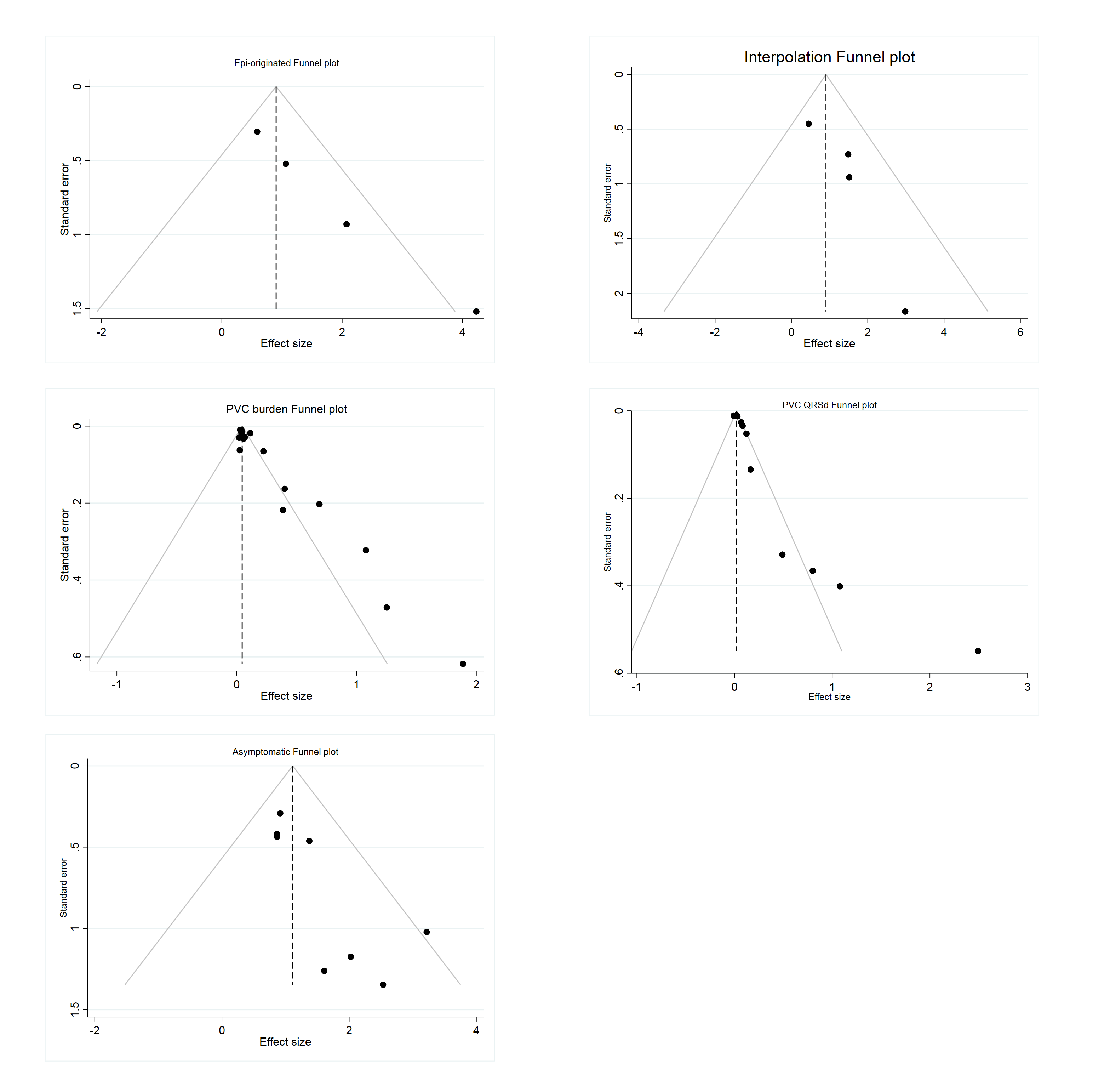

Supplement: Supplementary file 1 [file 2153-8174-25-9-327-s1.zip › Supplementary material 4 The funnel plots of factors predicting PVC-CMP.tif]

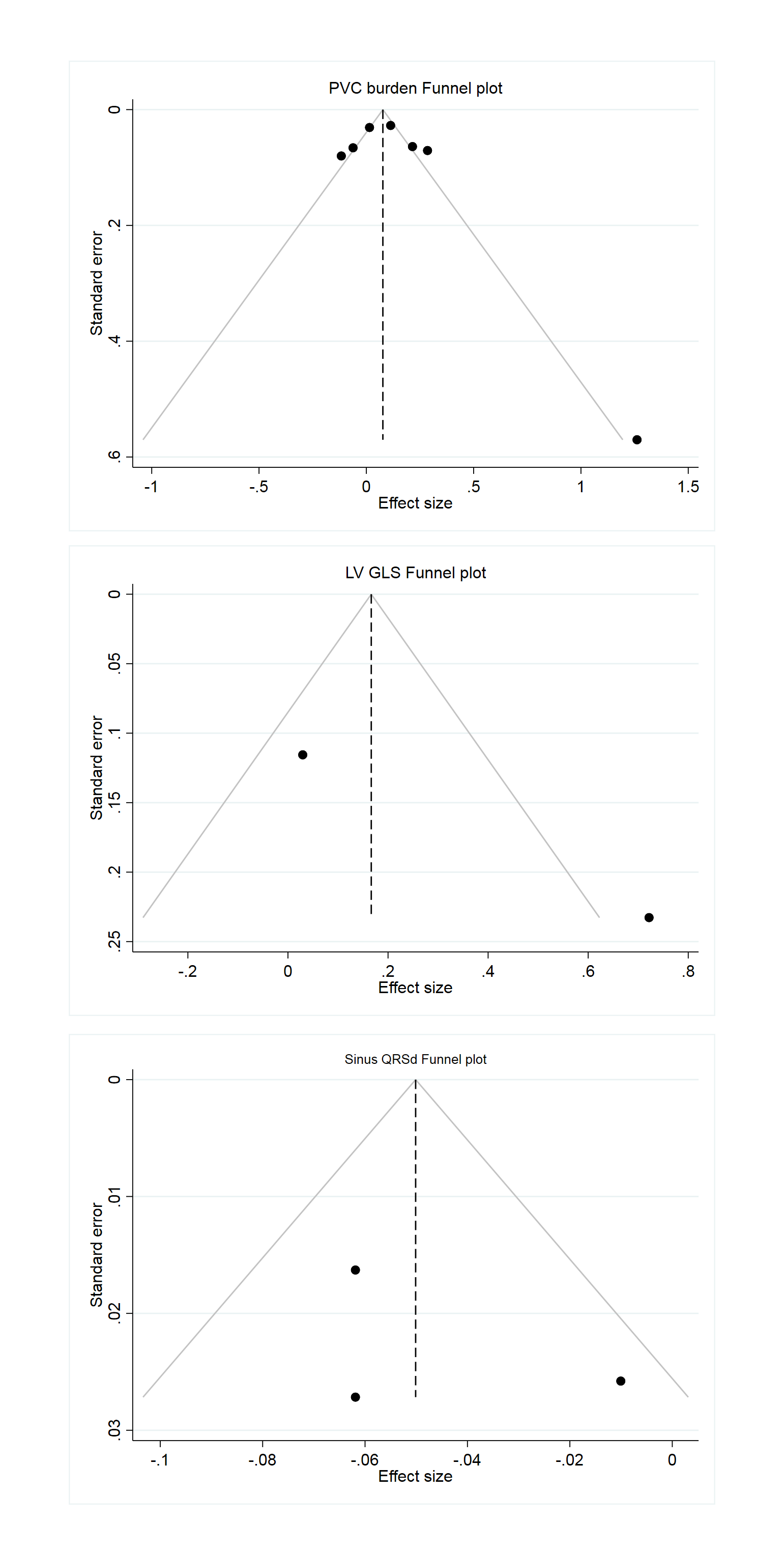

Supplement: Supplementary file 1 [file 2153-8174-25-9-327-s1.zip › Supplementary material 5 The funnel plots of factors associated with PVCs exacerbated LVSD.tif]
